# Chicago Medical Society: Stated Meeting March 1st

**Published:** 1886-05

**Authors:** 


### SOCIETY REPORTS.

---

CHICAGO MEDICAL SOCIETY.

*Stated Meeting, March 1st, 1886.*—THE PRESIDENT, C. T. PARKES, M. D., in the chair.

DR. P. C. JENSEN read the first paper of the evening, entitled

DIGESTION AND DYSPEPSIA.

The author entered into an elaborate discussion of the physiological processes of digestion, the departure from normal, and the treatment of gastritis, ulcer of stomach and atonic and nervous dyspepsia. Owing to the length of the paper the author was obliged to omit a large portion of it.

DR. J. FRANK thought that in the diagnosis of stomach disease pain under the left shoulder would not be a pathognomonic sign in case any cardiac trouble co-existed. It is well known that patients with cardiac disease complain of pain under the left shoulder. He had found that the majority of cases of dyspepsia are due to dilatation of the stomach and diseases of the pancreas.

PROFESSOR C. C. P. SILVA spoke of the omission, among the cases enumerated by the paper, of that common form of indigestion due to the excessive use of tea or coffee, especially when the tea or coffee has been boiled for a long time and drank after all the aromatic principles have been evaporated and there is left only the tannic acid, which precipitates pepsin. This is one of the most frequent causes of indigestion with ladies who abstain from cooking much and use only tea which is not freshly made, with bread and butter, taking such a lunch frequently.

DR. JENSEN, in concluding the discussion, said that he had found that in dyspeptic, as well as heart troubles, there is pain under the left shoulder. It is a symptom of dyspepsia when other affections can be excluded. As to dilatation of the stomach, it is often a cause of dyspepsia, and he had found it especially prevalent with beer drinkers who drink beer in large quantities, ten to twenty glasses a day. As to disease of the pancreas being a cause of dyspepsia, it is a very obscure organ and it is difficult to diagnosticate disease in it. In regard to long-drawn tea and coffee being an influential factor in the production of dyspepsia, he had observed that drinking too much liquid of any kind with the food has a very deleterious effect upon the digestion; reducing the quality of the gastric secretion, and thereby hindering the proper digestion of the food.

PROFESSOR J. H. ETHERIDGE read a report of

### TWO UNIQUE CASES OF VESICO-VAGINAL FISTULA.

In the first case the bladder appeared to be torn across from side to side about 1½ inches from the meatus urinarius, and the "flap" thus liberated was hermetically sealed to the posterior wall of the vagina. The cervix uteri was completely surrounded with adventitious connective tissue, in whose meshes was retained menstrual fluid.

In the second case, following a very protracted, severe labour, wherein the forceps had been fruitlessly used, and twelve hours later delivery was allowed to be spontaneously accomplished, the most extraordinary results ensued. *The*

*right ovary and uterus had completely disappeared by sloughing.* The left ovary remained.

THE PRESIDENT said he thought it a well-established fact that there is a possibility of an accidental expulsion of the uterus and its appendages occurring in connection with labour. During the past year there has been quite a discussion on the question of whether it is possible for such a thing to happen, and so far as the extract published in the *British Medical Journal* goes, it seems to prove that cases do occur in which, as far as we know, no interference with forceps or otherwise was made, and yet there was extrusion of the uterus and its appendages, and sufficient constriction thereof to produce sloughing and entire loss of these organs.

DR. F. M. WELLER remembered a case in which rupture of the bladder into the vagina occurred, and the only cause to be observed was some gravel-stones found in the bladder. He had no doubt that they were the result of vesico-vaginal fistula.

DR. R. TILLEY inquired if he had asked the physicians who attended these cases if forceps were used?

PROFESSOR ETHERIDGE knew nothing of the antecedent histories of these patients. The first was a Bohemian woman who spoke German indifferently, and it was difficult to get her history. The second patient was an American-born girl, and she gave pretty succinct history of her experience. She was in labour forty-eight hours, consultation being called at the end of thirty-six. Instruments were tried but failed to deliver her, and the physicians gave it up and went home. Finally pains came on and expulsion of the pelvic contents took place.

DR. H. P. NEWMAN read a report of

A CASE OF RUPTURED OVARIAN CYST, WITH SPECIMENS.

Mary H., unmarried; occupation, housework; age, twenty-five; Norwegian. Since her first menstruation, at thirteen years of age, the menses have been regular but scanty, accompanied by severe pain, necessitating her lying in bed the first day of each period. After coming to this country, four years ago, she seemed to suffer less in this respect, and while not strong, enjoyed a fair degree of health. In September of last year she had a mild attack of typhoid fever, convalescing at the end of the second week. During October she gained rapidly in flesh, and experienced less pain than usual at the menstrual molimen, as he afterward learned. In the second week of November following she was taken ill with peritonitis. It was during this attack, which was of several weeks' duration in the subacute and localized form, that his attention was first called to any abnormal condition of the pelvic organs. The tenderness of the abdomen and pelvic viscera rendered a thorough exploration impossible, and it was not until January that a satisfactory examination was made.

The patient first came to his office January 25th, when he found a tumor extending upward and a little to the right of the median line, from the lesser pelvic basin to midway between the umbilicus and symphysis pubis, and corresponding in size and general outline to the gravid uterus at the fourth month. Further investigation revealed fluctuation in the tumor, also that it was detached from the uterus; the latter being crowded to the left and back into the hollow of the sacrum. Although the fluid contents were not examined, the growth was pronounced a probable cyst of the right ovary, and the patient so advised. To any operative procedure the patient demurred, but promised to consider the matter and report at his office the following week. Nothing was heard from her until February 3rd, ten days later, when he was called to her house at about 6 P. M. He found the patient up, but complaining of pain and soreness of the abdomen, which was slightly tympanitic. Temperature 99½°, pulse slightly accelerated. With the use of an opiate and hot fomentations, the following morning he found her free from pain, with less tenderness over the abdomen, and no tympanitis. Pulse and temperature normal. He enjoined rest in bed, and directed that he should be called in case of further trouble.

On the night of February 6th he was summoned by the message that the patient was very low, and not expected to live through the night. He found the patient at 11 P. M. bolstered up in a chair, in a crouching attitude, with the thighs flexed upon the abdomen. The countenance bore such a pinched and anxious expression as gave striking evidence of grave peril. He was informed that following his visit on Thursday the patient had grown quite comfortable and free from pain, and contrary to his instructions sat up a large share of Friday. In the evening, while reading a letter, still sitting up in bed, she suddenly cried out that something had broken inside the abdomen. She suffered great distress, and was very much prostrated until 2 o'clock A. M., when she became more quiet. From this time she grew weaker, and had a very "bad feeling," as she described it, vomiting at intervals during the day, but suffering no particular pain. He was not called until late at night, twenty-four hours after the accident related. He found a rapid, feeble pulse of 140, skin moist, extremities cool, temperature 99° F., bowels tympanitic, but no pain, and little tenderness. She had passed no urine since the preceding night, and hardly a tablespoonful of dark fluid could be obtained by the catheter. A digital and bimanual examination revealed no change in the consistency and general outline of the tumor, as far as could be ascertained through the distended abdomen.

The gravity of the situation was explained to the friends, and an exploratory operation insisted upon as the only hope. As immediate consent could not be obtained, he advised a consultation. He thereby met Dr. A. B. Strong at ten o'clock that morning. Through the stimulants which had been freely administered during the night, the condition of the woman remained much the same. Suppression of the urine was still a marked symptom, abdominal tympanitis somewhat increased, temperature normal. The possibility of ruptured pelvic abcess, or intestinal perforation from fæcal impaction was considered, the doctor concurring with me in urging an immediate operation. As soon as arrangements could be made the patient was etherized and placed upon a table. He requested Dr. Strong to perform the operation. The incision through the abdominal wall was about three inches in length, midway between umbilicus and pubis in the median line. As soon as the peritoneal sac was opened there poured forth about three pints of purulent fluid. When this had been washed away, the tumor was readily recognized, filling the entire hypogastrium, its peritoneal surface engorged and discolored by the existing inflammation. Owing to the extensive adhesions, it became necessary to carry the original incision upward and through the umbilicus to a point two inches beyond, and downward to the symphisis. The fundus of the tumor was firmly attached along the under surface of the mesentery, making it necessary to ligate before removal; and, on closer inspection, a recently torn adhesion was observed, exposing a minute opening in the thinned wall of the sac, through which was oozing the purulent contents. In this connection it should be said that since her former attack of peritonitis the patient had complained of increasing pain on resuming the recumbent posture, a fact now easily accounted for by the extent and nature of the adhesions, which must have been thereby put upon the stretch. It is also probable that while sitting up in bed, movements of the body or abdominal viscera, together with the softening of adhesions by recent inflammation, had produced the rupture here found, and so perceptible to the patient. The complete separation of the remaining adhesions, and the removal of its growth with the right ovary and fallopian tube, was accomplished. The pedicle was tied with waxed silk, the ligature cut short and left within the pelvic cavity. All torn surfaces inclined to bleed were cauterized with a hot iron. The peritoneal cavity was washed out with hot water, dried with clean sponges, the wound closed, leaving a drainage tube in its lower angle, and the abdomen covered with antiseptic dressings.

Notwithstanding the free use of stimulants previously to the operation, and hypodermic injections of brandy toward its completion, within the next hour the woman's pulse became hardly perceptible at the wrist, and her immediate condition critical in the extreme. After the use of further injections of whisky and ammonia, and the application of bottles of hot water to the extremities, the patient rallied slightly and recognized those about her. From nine o'clock in the evening, however, she gradually sank, and died at eleven P.M. The abdomen was opened the next morning by Dr. Strong and the writer. Though covered with recent exudations of lymph, the peritoneal surfaces were of a better color, nor had bleeding occurred from any of the points of detachment.

The tumor as here presented is a unilocular, dermoid cyst, its purulent contents, about a pint and a half, resembling very much the fluid found in the pelvic cavity. It also contained a mass of fatty substance bound together with a quantity of hair. The right fallopian tube here attached, was uniformly enlarged and held a number of drops of pus. The left ovary, which was removed post-mortem, also contained a few small cysts. The uterus was normal both in size and appearance.

The points of interest are: first, the disparity between the temperature noted and the extreme inflammatory changes in the peritoneal cavity; second, the difficulty in differentiating between rupture of this cyst and a possible pelvic abcess; also the resemblence of the symptoms to those observed in a case of twisted pedicle, as reported to the society by our President, Dr. Parkes, at a recent meeting; and third, the liability of rupture of ovarian cysts even of small size, where inflammation occurs, constitutes additional arguments in favor of early operations.

DR. A. B. STRONG said he believed it was generally supposed by the profession that peritonitis is always accompanied by an elevation of temperature as indicated by the thermometer. He had recently seen a case in which a large quantity of pus was removed by section from the abdomen of a boy, in which the thermometer did not indicate any rise of temperature. These two cases seem to show that in purulent peritonitis the temperature is not always a practical aid in diagnosis, unless in a negative way.

THE PRESIDENT was inclined to think from some of the symptoms reported that this was a case of twisted pedicle, and that the rupture was caused by the twisting of the pedicle, which is quite often the result of the subsequent distension of the cyst walls; if it is weak at any point, it ruptures. Darkness in color is one noticeable change found in twisted pedicle, and if this was not a case of twisting, there was great resemblance in the symptoms and those of cases he had seen, viz., rapid distension of the abdomen, increased temperature, symptoms of peritonitis, and suppression of urine. In case the ruptured cyst was dependent upon twisted pedicle it might account for the separation of the adhesion, which was found. The mere fact of the movement of the body, which was advised against, might have precipitated a turn of the tumor upon itself, which led to the rupture by a diffusion of blood. He thought it a very interesting case and one calling attention to the necessity of insisting upon early interference in all cases where there is a supposed tumor of the abdomen, accompanied by the symptoms mentioned. And where the operation is done early there is very little difference in the fatality as compared with the operation done without peritonitis being present:

DR. NEWMAN said, in conclusion, that he did not think twisted pedicle was present in this case. The adhesions of the upper part and sides of the tumor were of such a nature that there could be but slight twisting of the base on the pedicle, and if it had occurred as a factor in separating the adhesions it must have returned to its normal position again, for the tumor, when found, occupied its usual position.

DR. STRONG said he was sure the pedicle was not twisted. There were two points of attachment to the tumor, one adhering to the base of the mesentery on the right side, the other to the sigmoid flexure, both old adhesions, and the points of attachment so located that it would be impossible for the pedicle to be twisted.

THE PRESIDENT wished to know how the dark color was accounted for.

DR. STRONG said that it looked at first very much like a uterine tumor, being thick and vascular. It was a light mahogany color, but was not such a color as is found in a strangulated gut.

PROFESSOR J. H. ETHERIDGE said, in reference to the lack of correspondence between the temperature as indicated by the thermometer and a high degree of inflammation, that the temperature will often be found in cases of peritonitis to be sub-normal. In cases of gonorrhœa in the female, complicated by pelvic peritonitis, physicians are often misled by the fact that the inflammation which produces great pain in the abdomen is not accompanied by a temperature which would lead him to suppose that peritonitis is present, thus causing him to erroneously rule out the possibility of there being peritonitis.

DR. G. W. WEBSTER exhibited a convenient

CASE OF ANTIDOTES FOR POISONS, WITH STOMACH TUBE.

He said that in his limited experience in the treatment of patients who had taken poison, either accidentally or otherwise, he had often found it difficult to procure the proper antidotes quickly enough and in a suitable form. It was this that led him to devise this case, intending at the time to have only one made for his own use. The case itself contains a pamphlet concerning poisons and their antidotes, a stomach tube, and the following drugs: ether, ammonia carbonate, nitrate of amyl, apomorphia, sulphate of atropia, brandy, camphor, animal charcoal, chloral hydrate, chloroform, digitaline, dialyzed iron, sulphate of iron, tr. of chloride of iron, mucilage, calcined magnesia, sulphate of morphia, iodide of potassium, liquor of potassæ, acetate of strychnia, chloride of sodium, sulphuric acid, tannic acid, sulphate of zinc. The atropia, morphia, apomorphia, strychnia and digitaline have been made up in compressed tablets and combined with soda so that they can be given hypodermically.

DR. J. FRANK exhibited a specimen of

DEGENERATED RIGHT KIDNEY, with a brief history of the case. Thirteen weeks ago, lithotrity was attempted on a man sixty-nine years old. The lithotrite broke, and the next day lithotomy was performed. The patient did well for about three weeks when he seemed to fail again, and pus appeared in the urine. Then he got better, and the pus disappeared. But a relapse came and he died. During the time he was voiding pus, Dr. Frank had made microscopic examinations to find casts, but was unsuccessful. There was total degeneration of the medullary substance of the kidney exhibited, while the other was full of renal calculi.

THE PRESIDENT remarked that the degeneration of the kidney was so great that tube cast formation would be impossible.

DR. R. TILLEY exhibited a sample of

### LANOLIN

which he obtained through favor from Frazer & Co., of New York. He said lanolin has lately been brought prominently before the medical public by the researches of Professor Oscar Liebreich, of Berlin, and its clinical applications by Liebreich and others. The substance exhibited, you will observe, is of a yellowish-brown color, and of a plastic consistency. The upper layer is darker than that immediately beneath. Its odor is slight, but *sui generis*. Liebreich says it should smell like wool. It is found in practically all the keratine tissues of the animal economy, but the commercial article is undoubtedly obtained from wool. The sample he supposed to be a mixture of equal parts of pure lanolin and water. Liebreich calls it a cholesterin fat, that is, a substance composed of fatty acid and cholesterin. The more commonly recognized animal fats being, of course, compounds of fatty acids and glycerine. He promises later to give us its exact chemical composition. The special point of interest about it to us as physicians, is, that it seems to be absorbed by the integument with much greater facility than the substances now in general use as bases for ointments. In the *British Medical Journal* of Feb. 13, 1886, a number of formulæ are given, being so far the result of Professor Liebreich's observations as to the most convenient method of associating it with other substances. He there refers to several clinical cases of interest, psoriasis, favus, and eczema.

He regards it as an excellent base for blue ointment. He claims that when a small quantity of a sublimate ointment of 1 in 1,000 made with lanolin is rubbed on the skin, that in a few minutes the characteristic metallic taste of mercury appears in the mouth, and from this and other observations concludes that all toxic agents should be used, when associated with lanolin, with great caution.

